# Age-related differences in the association between executive function and social responsiveness in autism spectrum disorder: a multi-method study

**DOI:** 10.3389/fpsyt.2026.1729973

**Published:** 2026-03-12

**Authors:** Jiarou Chen, Kaiyue Han, Xingxing Liao, Junzi Long, Xianna Wang, Yan Zhang, Weiwei Luo, Zhiqing Tang, Hao Zhang

**Affiliations:** 1 Rehabilitation Medicine Center, The Second Affiliated Hospital and Yuying Children’s Hospital, Wenzhou Medical University, Wenzhou, Zhejiang, China; 2 Beijing Boai Hospital, China Rehabilitation Research Center, Beijing, China; 3 School of Rehabilitation, Capital Medical University, Beijing, China; 4 Division of Brain Sciences, Changping Laboratory, Beijing, China; 5 China Autism Rehabilitation Research Center, Beijing Boai Hospital, China Rehabilitation Research Center, Beijing, China

**Keywords:** autism spectrum disorder, executive function, latent profile analysis, social responsiveness, working memory

## Abstract

**Background:**

Executive function (EF) deficits are a core cognitive feature of autism spectrum disorder (ASD) and are closely associated with social responsiveness. Previous research has primarily focused on children with ASD, whereas how specific executive components relate to social functioning in adults remains less clear. This study examined whether patterns of association between EF and social responsiveness differ between children and adults with and without ASD.

**Methods:**

Data were obtained from the Autism Brain Imaging Data Exchange II (ABIDE II), including 423 participants aged 8–23 years (ASD = 184; controls = 239). EF was evaluated using the Behavior Rating Inventory of Executive Function (BRIEF/BRIEF-A), and social responsiveness was assessed with the Social Responsiveness Scale (SRS). Covariates of age, sex, and full-scale IQ (FIQ) were controlled using entropy balancing in children and multiple regression in adults. Hierarchical regression, moderated mediation analysis, and latent profile analysis (LPA) were conducted to examine the moderation, mediation, and heterogeneity effects, respectively.

**Results:**

Across both child and adult samples, individuals with ASD exhibited significantly higher T-scores than controls on nearly all BRIEF and SRS subdomains after covariate adjustment (all adjusted p < 0.01), indicating widespread EF and social responsiveness impairments. Moderation analyses revealed no significant age group × EF interaction, indicating that the association between EF and social responsiveness was consistent across development. Mediation analysis revealed age-specific pathways, with EF broadly mediating social responsiveness in adults but showing more selective mediation in children. LPA identified four distinct subtypes, which were independent of age, sex, and FIQ.

**Conclusions:**

EF–social responsiveness associations were evident across development, but the functional contribution of specific executive components became more differentiated with age. Working memory showed greater relative prominence in adulthood. Latent profile analysis revealed heterogeneity in how executive difficulties align with social challenges, supporting developmentally informed assessment and clinical interpretation rather than direct treatment recommendations.

## 1 Introduction

Autism spectrum disorder (ASD) is a neurodevelopmental disorder characterized by persistent difficulties in social communication and interaction, together with restricted and repetitive patterns of behavior (1). Recent research indicates that the core challenges of ASD extend beyond social deficits and are closely linked to alterations in broader cognitive control systems (2). Specifically, executive function (EF) deficits are considered key contributors to social cognitive and behavioral abnormalities in ASD across the lifespan (3, 4). EF encompasses a set of higher-order cognitive processes that support goal-directed behavior, including inhibitory control, cognitive flexibility, working memory, emotional regulation, and self-monitoring (5, 6). These abilities collectively support planning, regulation, and adaptive behavior in complex social environments, and are therefore regarded as a crucial neurocognitive foundation for social deficits in ASD.

This study used the Behavior Rating Inventory of Executive Function (BRIEF) to assess everyday EF performance in children and adults with and without ASD. Based on theoretical frameworks and previous research, five BRIEF subscales (Inhibit, Shift, Working Memory, Emotional Control, and Monitor) were selected as the primary indicators because of their strong associations with social functioning in ASD, as well as their direct relevance to observable, everyday behaviors that are clinically relevant and measurable (7). Prior work has demonstrated robust associations between everyday executive functioning, including BRIEF-based profiles, and social skills in children with ASD, with multivariate analyses linking executive profiles to social behavior (8). Specifically, inhibition reflects the ability to control impulses and behavior, Shift measures cognitive flexibility in adapting to change, Working Memory involves the maintenance and manipulation of information, Emotional Control refers to the regulation of emotional responses, and Monitoring captures self-evaluation and oversight of performance (5, 9, 10). To measure core ASD symptoms and their relationship with EF, we administered the Social Responsiveness Scale (SRS). The SRS total score provides an index of overall social functioning, while the Social Communication and Autistic Mannerisms (later referred to as Restricted/Repetitive Behaviors) subscales map onto ASD’s two core domains: social-communication deficits and restricted/repetitive behaviors. These three indices were selected because they capture overall social impairment as well as the two diagnostically central symptom dimensions of ASD, and are commonly used as primary outcomes in studies examining cognitive–behavioral associations in ASD (11, 12).

A growing body of research has demonstrated that individuals with ASD exhibit deficits across multiple EF domains, which are closely linked to impairments in social responsiveness (7, 8). Inhibitory control and emotional control are key regulatory components of social interaction: the former supports suppression of inappropriate responses, while the latter facilitates emotional stability. Children with ASD often show heightened emotional reactivity, impulsive behavior, and social withdrawal, whereas adults with ASD commonly show emotional dysregulation and difficulties with inhibitory control (13–15).Consistent deficits in cognitive flexibility have been observed in individuals with ASD across different age groups, contributing not only to social interaction difficulties but also to increased repetitive and rigid behaviors (16, 17). Deficits in working memory, particularly in the updating and manipulation of information, are consistently reported in individuals with ASD and may further impair social functioning (18, 19). Deficits in task and self-monitoring can impede the monitoring of one’s behavior and the integration of external feedback, potentially leading to impaired social adaptation (20). Furthermore, deficits across multiple executive function domains in individuals with ASD have been associated with broader behavioral symptoms, including repetitive behaviors and difficulties in social interaction (21, 22). Neuroimaging studies further corroborate these behavioral findings, revealing that executive function deficits in ASD are associated with atypical neural activation patterns, which contribute to social communication difficulties and repetitive behaviors (23, 24). Collectively, these findings highlight heterogeneous contributions from distinct EF subdomains to social functioning that vary across development (25), suggesting that EF–social associations may vary across developmental stages (26).

In contrast to the extensive body of research on children, empirical evidence elucidating the relationship between EF and social functioning in adults with ASD remains limited (27). Moreover, the moderating role of age in EF-social associations and the potential mediating role of EF in the link between diagnosis and social abilities across development remain unclear. In addition, ASD is characterized by substantial heterogeneity, and person-centered approaches (e.g., latent profile analysis; LPA) may help characterize individual differences in the alignment between executive difficulties and social challenges, with potential implications for assessment and clinical formulation. Toward this goal, we adopted a cross-age design including both children and adults with and without ASD to examine the associations between five core EF subdomains (inhibition, shifting, emotional control, working memory, and monitoring) and social responsiveness as measured by the SRS total score and its social communication and restricted/repetitive behavior (mannerisms) subscales. Specifically, this study aimed to: (1) compare EF and social responsiveness between ASD and control participants within child and adult groups; (2) test whether age group moderates EF-social associations; (3) examine whether EF mediates the relationship between ASD diagnosis and social functioning, and whether this mediation is moderated by age; and (4) identify latent subgroups based on EF and social characteristics using LPA. We hypothesized that individuals with ASD would exhibit higher levels of EF difficulties and social impairments across all dimensions. Furthermore, EF deficits are expected to be associated with greater social symptom severity, and these associations may vary by age group. By directly comparing association patterns across children and adults, this study aims to clarify developmental differences in how specific executive components relate to social responsiveness and to provide a more precise characterization of EF–social organization and heterogeneity in ASD, with implications for developmentally informed assessment and interpretation rather than treatment prescription.

## 2 Methods

### 2.1 Participants

Data for this study were obtained from the Autism Brain Imaging Data Exchange II (ABIDE II; http://fcon_1000.projects.nitrc.org/indi/abide/). Participants were included if they met the following criteria: classified as children (8–15 years) or adults (18–23 years), and had complete behavioral assessments available, including the BRIEF/BRIEF-A and the SRS/SRS-2 parent- or informant-report forms. Participants with missing data on age, sex, or full-scale IQ (FIQ), extreme outliers (> 3 SD from the sample mean) on any BRIEF or SRS subscale, or FIQ below 80 were excluded. The final sample comprised 423 participants drawn from five ABIDE II sites, including 184 individuals with ASD (134 children and 50 adults) and 239 non-ASD controls (190 children and 49 adults).

Data used in this study were obtained from the Autism Brain Imaging Data Exchange II (ABIDE II) and were processed in accordance with the official ABIDE II quality control procedures. Contributing sites confirmed approval by their local institutional review board/ethics committee for the original data collection and retrospective sharing of fully de-identified datasets, and informed consent, when applicable, was obtained from participants or their legal guardians. The present study is a secondary analysis of de-identified data and was conducted in accordance with applicable regulations and institutional requirements, and did not require additional local ethical approval.

### 2.2 Measurements

#### 2.2.1 Executive function

EF was assessed using the BRIEF to assess everyday EF in naturalistic settings. The BRIEF Parent Form was used for child participants and the BRIEF-A Informant Form was used for adult participants, both of which are informant-reported measures. Five core subdomains were included in the present analyses: Inhibit, Shift, Emotional Control, Working Memory, and Monitor. Raw subscale scores were converted to standardized T-scores (M = 50, SD = 10) based on normative data, with higher scores indicating greater executive dysfunction (9, 28).

#### 2.2.2 Social responsiveness

Social responsiveness was measured using the SRS. The SRS Child Version was used for child participants and the SRS-2 Adult Version was used for adult participants; all versions are informant-reported. The SRS assesses difficulties in social cognition, social communication, social motivation, and restricted and repetitive behaviors. Three indices were included in the analyses: Social Communication, Mannerisms, and the Total Score. Raw scores were converted to T-scores (M = 50, SD = 10), with higher scores reflecting greater impairment in social functioning (29, 30).

#### 2.2.3 Full-scale intelligence quotient

FIQ data were obtained from the standardized cognitive assessments provided in the ABIDE II dataset. Each site administered age-appropriate versions of the Wechsler Intelligence Scales (e.g., WISC-IV/WISC-V or WAIS-IV), all of which are well-validated measures of intellectual functioning (31). FIQ was included as a covariate to account for potential influences of general cognitive ability on EF and social responsiveness.

### 2.3 Statistical analysis

All statistical analyses were conducted using Stata 18.0 (StataCorp LLC, College Station, TX, USA). The normality of all continuous variables was assessed using the Shapiro-Wilk test within each age group (children, adults) and diagnostic group (ASD, Control). Normally distributed data were presented as mean ± standard deviation and analyzed using two-sample t-tests; non-normally distributed data were presented as median (interquartile range, IQR) and analyzed using the Mann-Whitney U test. Categorical variables were compared using the chi-square (χ²) test, or Fisher’s exact test, as appropriate. To account for multiple comparisons across subdomains, Bonferroni correction was applied where applicable. A two-tailed p-value < 0.05 was considered statistically significant.

To account for potential confounding by age, sex, and FIQ, we applied different strategies for children and adults. In the child group, which had a larger sample size but greater covariate imbalance, entropy balancing was used to reweight the sample and achieve covariate balance (32). E-value analyses were conducted to quantify the minimum strength of unmeasured confounding that could explain away observed associations. In the adult group, given the smaller sample size, covariate effects were controlled using multivariable regression models, as matching or weighting could lead to estimation instability in sparse data settings.

Furthermore, we fitted multiple regression models that included an interaction term between EF and age group to examine whether the association between EF and social responsiveness was moderated by age group. All models controlled for sex, age, and FIQ (33, 34). A moderated mediation analysis was subsequently conducted to test whether EF mediated the association between diagnostic group (ASD vs. control) and social responsiveness, and whether this indirect effect varied by age group. The model was estimated using bootstrap resampling (1000 iterations) to obtain bias-corrected 95% confidence intervals for the indirect effect (35, 36).

Finally, an LPA was conducted using T-scores from the five BRIEF subdomains and three SRS dimensions. Competing models (1–4 classes) were evaluated based on the Akaike Information Criterion (AIC) and Bayesian Information Criterion (BIC). The optimal model was selected based on a combination of statistical fit indices and theoretical interpretability (37).

These analytic strategies were designed to maximize comparability across age groups, accounting for sample size differences and minimizing bias from covariate imbalance. Detailed statistical procedures are provided in the **Supplementary Methods**.

## 3 Results

All behavioral scores were standardized T-scores adjusted for normative data; therefore, the reported findings reflect relative group-level differences within each age cohort rather than absolute cross-age comparisons.

### 3.1 Descriptive statistics and covariate adjustment

In both the child and adult samples, participants with ASD showed significantly higher T-scores than controls across all measures of EF and social responsiveness (all unadjusted p < 0.01; **Table 1**). Demographic comparisons indicated no significant between-group differences in age for the child sample and no significant differences in FIQ for the adult sample. In the child sample, entropy balancing produced satisfactory covariate balance (effective sample size [ESS] = 117; standardized mean differences [SMDs] < 0.10 for all covariates; **Supplementary Tables 1**-**3** and **Supplementary Figure 1**). After weighting, the ASD group continued to show significantly higher T-scores than controls across all BRIEF and SRS subdomains. Notably, effect sizes were consistently larger for the SRS measures than for the BRIEF (EF) measures. After Bonferroni correction for multiple comparisons (α_adjusted = 0.00625), all differences remained highly significant (p < 0.001 for all) with large effect sizes (Cohen’s d > 1.40; **Table 2**). E-value analyses indicated that the observed associations were robust to unmeasured confounding. Among the SRS domains, the largest group difference was observed for SRS total score (adjusted mean difference B = 35.52, 95% CI [33.19, 37.85], d = 1.76), followed by the communication and mannerisms subscales (adjusted B > 33, d > 1.68). For EF, the greatest deficits were noted in shifting and working memory (adjusted B > 26, d > 1.55).

**Table 1:** Executive function and social responsiveness: comparing demographic and clinical profiles of ASD and control groups.

	Children			Adults		
Variable	ASD (n = 134)	Control (n =190)	p-value	ASD (n = 50)	Control (n = 49)	p-value
Demographics						
Age	11.00 (10.54, 13.63)	12.00 (10.90, 12.64)	0.09	22.00 (21.00, 22.08)	19.25 (19.00, 20.00)	<0.01
FIQ	105.00 (94.00, 114.00)	114.00 (106.00, 122.00)	<0.01	117.00 (108.00, 129.00)	124.00 (113.00, 132.00)	0.12
Males, n (%)	110 (82.09%)	137 (72.11%)	0.04	43 (86.00%)	24 (48.98%)	<0.01
Clinical tests						
SRS						
Total	80.00 (71.00, 88.00)	42.00 (40.00, 46.00)	<0.01	75.00 (67.00, 86.00)	42.00 (40.00, 45.00)	<0.01
Communication	76.00 (67.00, 86.00)	42.00 (39.00, 46.00)	<0.01	70.00 (64.00, 85.00)	43.00 (38.00, 46.00)	<0.01
Mannerisms	79.00 (70.00, 90.00)	44.00 (41.00, 46.00)	<0.01	76.00 (67.00, 90.00)	42.00 (41.00, 46.00)	<0.01
BRIEF						
Inhibit	64.00 (56.00, 71.00)	42.00 (40.00, 48.00)	<0.01	60.00 (50.00, 66.00)	42.00 (40.00, 49.00)	<0.01
Shift	70.00 (63.00, 77.00)	41.00 (39.00, 47.00)	<0.01	67.00 (59.00, 77.00)	43.00 (39.00, 49.00)	<0.01
Emotional Control	63.00 (55.00, 72.00)	42.00 (38.00, 47.00)	<0.01	58.00 (51.00, 67.00)	47.00 (38.00, 51.00)	<0.01
Working Memory	69.00 (63.00, 74.00)	43.00 (39.00, 49.00)	<0.01	63.50 (56.00, 71.00)	46.00 (40.00, 50.00)	<0.01
Monitor	66.00 (60.00, 73.00)	43.00 (37.00, 50.00)	<0.01	62.00 (53.00, 72.00)	41.00 (36.00, 50.00)	<0.01

ASD, autism spectrum disorder; SRS, Social Responsiveness Scale; BRIEF, Behavior Rating Inventory of Executive Function; FIQ, full-scale IQ. Values are presented as median [Q1, Q3]. Group comparisons were performed using the Mann–Whitney U test (two-tailed) for continuous variables and the chi-square test (χ^2^) (or Fisher’s exact test when appropriate) for categorical variables.

**Table 2:** Comparison of core measures of executive function and social responsiveness between ASD and control groups after entropy balancing weighting.

Variable	Control, mean (SE)	ASD, mean (SE)	B(SE)	95%CI	p-value	Cohen’s d	E-value
SRS-Total	43.11 (0.50)	78.63 (1.08)	35.52 (1.18)	[33.19, 37.85]	**<0.001**	1.76	7.3 × 10^11^
SRS-Communication	43.15 (0.49)	76.40 (1.10)	33.25 (1.21)	[30.87, 35.64]	**<0.001**	1.72	6.9 × 10^10^
SRS-Mannerisms	44.31 (0.55)	80.28 (1.38)	35.97 (1.46)	[33.10, 38.84]	**<0.001**	1.68	5.6 × 10^9^
Inhibit	44.23 (0.53)	63.88 (1.07)	19.65 (1.20)	[17.28, 22.01]	**<0.001**	1.42	5.6 × 10^9^
Shift	43.38 (0.57)	69.85 (1.13)	26.47 (1.24)	[24.03, 28.90]	**<0.001**	1.59	2.9 × 10^8^
Emotional Control	43.36 (0.58)	62.38 (1.10)	19.02 (1.20)	[16.65, 21.38]	**<0.001**	1.4	1.8 × 10^6^
Working Memory	45.17 (0.65)	67.45 (0.92)	22.28 (1.13)	[20.06, 24.51]	**<0.001**	1.55	6.1 × 10^7^
Monitor	44.70 (1.01)	65.30 (0.96)	20.60 (1.37)	[17.91, 23.29]	**<0.001**	1.41	8.9 × 10^5^

ASD, Autism Spectrum Disorder; SE, Standard Error; B, unstandardized regression coefficient (representing the adjusted mean difference between groups);SE, standard errors; CI, Confidence Interval.β represents the mean difference (ASD - Control). Cohen’s d denotes effect size (where |d| > 0.8 represents a large effect). E-value indicates the minimum strength of association an unmeasured confounder would need to have to explain away the observed effect. Entropy balancing Weighting was applied using gender, age, and FIQ as covariates. Bold p-values indicate significance after Bonferroni correction for 8 comparisons (corrected α = 0.00625).

In the adult sample, multivariable linear regression models adjusting for sex, age, and FIQ showed that the ASD group had significantly higher T-scores than controls for most BRIEF and SRS measures (Bonferroni-corrected α = 0.00625; **Table 3**), with the exceptions of emotional control and monitoring, which did not survive correction. As shown in **Table 4**, regression analyses indicated that the diagnostic group significantly predicted all SRS and BRIEF subdomains (all p < 0.05). The largest unstandardized effect was observed for SRS mannerisms (B = 33.90), followed by shift (B = 20.20) and inhibition (B = 19.19), both also exhibited strong standardized coefficients (β = 0.655 and β = 0.837, respectively; **Supplementary Table 4**). In terms of variance uniquely attributable to diagnostic group, the effect was largest for SRS mannerisms (partial η² = 0.28) and smallest for monitoring (partial η² = 0.04; **Supplementary Table 5**). Among covariates, sex significantly predicted SRS mannerisms (B = 5.93, p < 0.05) and working memory (B = 4.49, p < 0.05), while age was a significant predictor only for monitoring (B = 4.08, p < 0.05). FIQ was not significant in any of the models (**Table 4**). Model diagnostics indicated no evidence of problematic multicollinearity (all variance inflation factors [VIFs] < 5; **Supplementary Table 6**), and the models demonstrated adequate to high explanatory power (adjusted R² = 0.29-0.70).

**Table 3:** Comparison of adjusted mean scores on executive function and social responsiveness between ASD and control groups in adults.

Variable	ASD, adjusted mean (95% CI)	ASD, Standard error	Control, adjusted mean (95% CI)	Control, Standard error	Mean difference (95% CI)	p-value
SRS-Total	73.50 (68.24, 78.77)	2.65	45.20 (39.85, 50.55)	2.70	28.30 (18.65, 37.96)	**<0.001**
SRS-Communication	70.79 (65.59, 75.98)	2.61	45.99 (40.72, 51.27)	2.66	24.79 (15.27, 34.32)	**<0.001**
SRS-Mannerisms	78.23 (72.23, 84.23)	3.02	44.33 (38.23, 50.43)	3.07	33.90 (22.89, 44.91)	**<0.001**
Inhibit	62.15 (57.68, 66.63)	2.25	42.97 (38.42, 47.52)	2.29	19.19 (10.98, 27.40)	**<0.001**
Shift	65.85 (60.71, 70.98)	2.59	45.65 (40.42, 50.87)	2.63	20.20 (10.78, 29.62)	**<0.001**
Emotional Control	58.25 (53.30, 63.21)	2.50	47.21 (42.17, 52.25)	2.54	11.04 (1.95, 20.14)	0.018
Working Memory	60.77 (56.44, 65.10)	2.18	49.36 (44.95, 53.76)	2.22	11.41 (3.46, 19.36)	**0.005**
Monitor	57.27 (52.42, 62.12)	2.44	48.13 (43.20, 53.06)	2.48	9.14 (0.24, 18.03)	0.044

ASD, Autism Spectrum Disorder; CI, Confidence Interval. Adjusted means were derived from multiple regression models controlling for gender, age, and FIQ. All mean differences (ASD - control) were statistically significant at p < 0.05. Bold p-values indicate significance after Bonferroni correction for 8 comparisons (corrected α = 0.00625).

**Table 4:** Multiple linear regression analyses predicting executive function and social responsiveness scores in the adult sample.

Variable	ASD group (Reference: Control group)		Male (Reference: Female)	Age	FIQ	Constant	Adjust R²
	B(SE)	Partial eta-squared	B(SE)	B(SE)	B(SE)	B(SE)	
SRS-Total	28.30^***^ (4.86)	0.26	3.82(2.60)	2.83(1.83)	-0.00(0.08)	-14.02 (36.14)	0.697
SRS-Communication	24.79^***^ (4.80)	0.22	4.52(2.56)	3.09(1.81)	0.04(0.08)	-24.01 (35.65)	0.658
SRS-Mannerisms	33.90^***^ (5.55)	0.28	5.93^*^(2.96)	1.56(2.09)	-0.03(0.09)	14.54 (41.21)	0.667
Inhibit	19.19^***^ (4.13)	0.19	4.05(2.21)	-2.07(1.56)	-0.03(0.07)	87.36^**^ (30.73)	0.337
Shift	20.20^***^ (4.75)	0.16	0.64(2.53)	1.16(1.79)	-0.00(0.08)	22.23 (35.27)	0.518
Emotional Control	11.04^*^ (4.58)	0.06	-0.11(2.45)	1.24(1.72)	0.07(0.07)	13.97 (34.03)	0.288
Working Memory	11.41^**^ (4.00)	0.08	4.49^*^(2.14)	2.86(1.51)	-0.08(0.06)	-1.17 (29.75)	0.473
Monitor	9.14^*^ (4.48)	0.04	2.72(2.39)	4.08^*^(1.69)	-0.04(0.07)	-32.28 (33.30)	0.438

ASD, Autism Spectrum Disorder; SRS, Social Responsiveness Scale; B, unstandardized regression coefficients; SE, standard errors. ^*^p < 0.05, ^**^p < 0.01, ^***^p < 0.001. The reference group for the analysis is the Control group for Group comparison and Female for gender comparison.

### 3.2 Moderation analysis

We tested whether age group moderated EF-social responsiveness associations by fitting multiple linear regression models with EF × age group interaction terms. Model diagnostics indicated no problematic multicollinearity (all VIFs < 2.0; **Supplementary Table 7**), and model fit was acceptable (adjusted R² = 0.211-0.451, all p < 0.001; **Table 5**). Within-group simple slope analyses revealed significant positive associations between each EF domain and SRS outcomes in both children and adults (all p < 0.01; **Table 5**). In uncorrected analyses, the working memory × age group interaction reached nominal significance for all SRS dimensions (interaction p < 0.05); the unstandardized slope for working memory predicting SRS total score was steeper in adults (B = 1.01, SE = 0.16, p < 0.01) than in children (B = 0.56, SE = 0.10, p < 0.01). A marginally significant group difference was noted for inhibition predicting mannerisms (adults B = 0.98; children B = 0.56, interaction p = 0.06). No significant interaction effects were found for shifting, emotional control, or monitoring.

**Table 5:** Moderating effect analysis of age group on the relationship between executive function and social function.

BRIEF	SRS	Interaction effect, B (SE)	Interaction effect, p	Simple slope (Child), B (SE)	Simple slope (Adult), B (SE)	Adjust R^2^
Inhibit	Total	0.20 (0.18)	0.27	0.50 (0.09) ^***^	0.70 (0.16) ^***^	0.233
	Communication	0.12 (0.18)	0.52	0.51 (0.09) ^***^	0.63 (0.16) ^***^	0.231
	Mannerisms	0.42 (0.22)	0.06	0.56 (0.10) ^***^	0.98 (0.19) ^***^	0.234
Shift	Total	0.02 (0.13)	0.88	0.72 (0.07) ^***^	0.74 (0.11) ^***^	0.451
	Communication	-0.08 (0.14)	0.60	0.68 (0.08) ^***^	0.61 (0.12) ^***^	0.373
	Mannerisms	-0.08 (0.16)	0.63	0.89 (0.09) ^***^	0.81 (0.13) ^***^	0.436
Emotional Control	Total	0.07 (0.17)	0.66	0.60 (0.08) ^***^	0.67 (0.15) ^***^	0.289
	Communication	-0.05 (0.18)	0.78	0.54 (0.09) ^***^	0.49 (0.16) ^**^	0.218
	Mannerisms	0.02 (0.21)	0.93	0.69 (0.10) ^***^	0.71 (0.18) ^***^	0.244
Working Memory	Total	0.46 (0.18)	0.014^*^	0.56 (0.10) ^***^	1.01 (0.16) ^***^	0.302
	Communication	0.43 (0.19)	0.023^*^	0.53 (0.10) ^***^	0.96 (0.16) ^***^	0.277
	Mannerisms	0.47 (0.23)	0.044^*^	0.57 (0.12) ^***^	1.04 (0.20) ^***^	0.211
Monitor	Total	0.25 (0.17)	0.14	0.68 (0.09) ^***^	0.93 (0.15) ^***^	0.357
	Communication	0.27 (0.18)	0.12	0.65 (0.09) ^***^	0.92 (0.15) ^***^	0.337
	Mannerisms	0.15 (0.22)	0.50	0.71 (0.12) ^***^	0.87 (0.19) ^***^	0.242

SRS, Social Responsiveness Scale; BRIEF, Behavior Rating Inventory of Executive Function; B, unstandardized regression coefficient; SE, standard error. ^***^p < 0.001, ^**^p < 0.01, ^*^p < 0.05(uncorrected); Bonferroni correction for 15 tests (adjusted α = 0.0033). All models controlled for gender and FIQ.

### 3.3 Mediation analysis

Moderated mediation analyses revealed age-dependent mediation pathways linking diagnostic group to social responsiveness via executive functioning. In adults, each of the five EF components demonstrated significant indirect effects on all three SRS domains (bootstrap, 1,000 samples; bias-corrected 95% CIs did not include zero; **Table 6**), indicating broad and consistent mediation. Conversely, mediation in the child group was more selective: shifting and emotional control showed significant indirect effects across all SRS outcomes, inhibition mediated SRS communication scores specifically, and monitoring mediated SRS total and communication scores. Working memory did not produce any significant indirect effects. Direct comparisons of indirect effects across age groups indicated that the indirect effect of working memory was significantly stronger in adults than in children for all SRS outcomes (total: indirect B = -7.05, 95% CI [-13.71, -0.24]; communication: B = -7.11, 95% CI [-13.71, -0.53]; mannerisms: B = -8.19, 95% CI [-15.94, -0.47]). A nominal group difference was also observed for shifting on communication (ΔB_indirect = 2.73, p = 0.032), albeit with a confidence interval that included zero. No other indirect effects differed significantly. Importantly, direct effects of diagnostic group on SRS outcomes remained significant after accounting for EF mediators.

**Table 6:** Mediation effects of executive functions on social responsiveness across age groups.

BRIEF	SRS	Child indirect effect	Adult indirect effect	Indirect effect difference	Direct effect	Total effect
Inhibit	Total	-7.69 [-16.30, 0.10]	**-7.55 [-9.68, -5.59]**	0.13 [-6.89, 8.04]	**-28.64 [-35.67, -21.54]**	**-36.26 [-41.36, -31.01]**
	Communication	**-9.32 [-18.12, -1.65]**	**-7.86 [-10.11, -5.79]**	1.45 [-4.98, 8.76]	**-26.82 [-33.90, -20.05]**	**-35.41 [-40.41, -30.02]**
	Mannerisms	-4.22 [-13.80, 5.37]	**-8.14 [-11.25, -5.15]**	-3.92 [-11.60, 3.38]	**-28.54 [-37.24, -20.25]**	**-34.72 [-40.07, -28.62]**
Shift	Total	**-13.84 [-22.42, -6.18]**	**-13.64 [-16.46, -11.10]**	0.20 [-5.71, 6.96]	**-23.23 [-30.74, -15.79]**	**-36.97 [-42.03, -32.05]**
	Communication	**-15.43 [-24.20, -7.94]**	**-12.70 [-15.59, -10.07]**	2.73^*^ [-2.80, 9.89] (p=0.032)	**-21.47 [-28.81, -14.02]**	**-35.54 [-40.66, -30.17]**
	Mannerisms	**-17.66 [-28.75, -8.09]**	**-16.34 [-20.06, -12.82]**	1.32 [-5.85, 10.12]	**-18.10 [-27.08, -8.98]**	**-35.10 [-40.47, -29.16]**
Emotional Control	Total	**-10.57 [-18.19, -4.14]**	**-8.63 [-10.53, -6.77]**	1.94 [-3.63, 8.41]	**-26.69 [-33.18, -20.60]**	**-36.28 [-41.21, -31.01]**
	Communication	**-10.93 [-18.61, -4.29]**	**-7.63 [-9.70, -5.66]**	3.30 [-2.27, 9.79]	**-26.30 [-33.06, -19.99]**	**-35.58 [-40.65, -30.16]**
	Mannerisms	**-12.18 [-21.53, -3.90]**	**-9.65 [-12.43, -6.99]**	2.53 [-4.06, 10.56]	**-23.69 [-31.10, -16.29]**	**-34.60 [-40.16, -28.40]**
Working Memory	Total	-1.05 [-9.32, 7.21]	**-8.10 [-10.72, -5.37]**	**-7.05 [-13.71, -0.24]**	**-32.78 [-40.82, -25.02]**	**-37.36 [-42.16, -32.38]**
	Communication	-0.39 [-8.69, 7.75]	**-7.50 [-10.08, -4.78]**	**-7.11 [-13.71, -0.53]**	**-32.73 [-40.84, -24.79]**	**-36.67 [-41.38, -31.52]**
	Mannerisms	0.71 [-8.96, 10.16]	**-7.47 [-10.58, -4.40]**	**-8.19 [-15.94, -0.47]**	**-32.60 [-41.32, -23.58]**	**-35.98 [-41.48, -29.85]**
Monitor	Total	**-7.25 [-13.59, -1.55]**	**-8.90 [-10.80, -7.14]**	-1.65 [-6.61, 3.69]	**-29.21 [-35.57, -22.57]**	**-37.28 [-42.33, -31.94]**
	Communication	**-5.96 [-11.96, -0.29]**	**-8.33 [-10.31, -6.64]**	-2.37 [-7.10, 2.75]	**-29.57 [-35.91, -22.97]**	**-36.71 [-41.60, -31.53]**
	Mannerisms	-6.03 [-14.27, 1.57]	**-8.17 [-10.87, -5.58]**	-2.14 [-8.26, 4.44]	**-28.74 [-37.20, -20.64]**	**-35.84 [-41.58, -29.54]**

SRS, Social Responsiveness Scale. Indirect, direct, and total effects are unstandardized regression coefficients (B). 95% confidence intervals are bias-corrected and based on 1,000 bootstrap samples. Bold values indicate significant effects (95% confidence interval does not include zero). Covariates: All models included gender and FIQ as covariates. *p < 0.05 indicates significance (Bonferroni-corrected).

### 3.4 Latent profile analysis

Results of the latent profile analysis supported the four-class solution based on both theoretical interpretability and statistical fit. The four-class model demonstrated the lowest AIC (3472.41) and BIC (3610.65) values in **Supplementary Table 8**, as well as excellent classification accuracy. Classification certainty for the selected model was high (mean posterior probabilities = 0.95-0.97). The class proportions were as follows in **Supplementary Table 9**: Class 1 (low symptoms, n = 39, 21.2%), Class 2 (mild-moderate, n = 67, 36.4%), Class 3 (moderate-severe, n = 67, 36.4%), and Class 4 (severe, n = 11, 6.0%). Significant graded differences in SRS total score were observed across classes (ANOVA: F (3,180) = 290.15, p < 0.001, η² = 0.83), with all pairwise comparisons remaining significant after Bonferroni correction in **Table 7**. Effect sizes for pairwise contrasts were uniformly very large (Cohen’s d range = 2.39-6.02; **Supplementary Table 10**), reflecting strong between-class differences. This graded pattern extended across BRIEF and SRS subscales (all p < 0.001; η² range = 0.28-0.83), with the largest class differences observed for social mannerisms (η² = 0.69) and cognitive shifting (η² = 0.50).Crucially, classes did not differ significantly in age, sex, or FIQ (all p > 0.05; **Table 8**), suggesting that the identified profiles reflect symptom and EF heterogeneity rather than demographic confounds.

**Table 7:** Executive function and social responsiveness scores by latent class.

Variable	Class 1 (n = 39)	Class 2 (n = 67)	Class 3 (n = 67)	Class 4 (n = 11)	F-value	p-value	η²
SRS-Total	60.62 ± 6.47	73.87 ± 4.95	87.82 ± 4.67	103.27 ± 9.05	290.15	<0.001	0.83
SRS-Communication	59.49 ± 7.06	71.00 ± 5.97	85.19 ± 6.55	101.55 ± 6.80	199.72	<0.001	0.77
SRS-Mannerisms	62.67 ± 9.43	74.66 ± 8.95	89.55 ± 8.35	114.36 ± 13.23	130.73	<0.001	0.69
Inhibit	53.05 ± 10.40	60.18 ± 9.84	68.18 ± 10.84	77.18 ± 10.29	26.01	<0.001	0.30
Shift	54.31 ± 9.42	68.24 ± 9.17	75.34 ± 9.34	88.36 ± 8.44	59.56	<0.001	0.50
Emotional Control	51.72 ± 10.07	60.75 ± 10.00	65.60 ± 11.46	77.45 ± 6.25	23.66	<0.001	0.28
Working Memory	55.87 ± 10.76	66.69 ± 8.53	70.58 ± 9.06	75.18 ± 8.94	24.7	<0.001	0.29
Monitor	52.05 ± 9.90	64.30 ± 9.38	69.72 ± 6.80	73.55 ± 8.02	39.73	<0.001	0.40

SRS, Social Responsiveness Scale. All values represent mean ± standard deviation. All F-values have degrees of freedom (3,180). η² (eta squared) is a measure of effect size indicating the proportion of variance in the dependent variable accounted for by group differences. (η² ≥ 0.01 small, ≥ 0.06 medium, ≥ 0.14 large.

**Table 8:** Demographic and clinical characteristics by latent class.

Characteristic	Class 1 (n = 39)	Class 2 (n = 67)	Class 3 (n = 67)	Class 4 (n = 11)	Statistic	p-value
Age, Years	15.83 ± 4.97	14.14 ± 4.68	13.62 ± 4.60	13.21 ± 5.67	F = 1.99	0.117
FIQ	112.03 ± 15.87	107.99 ± 14.57	107.85 ± 15.91	113.18 ± 15.94	F = 0.99	0.400
Sex, Male/Female	57 (85.1%)/10 (14.9%)	52 (77.6%)/15 (22.4%)	8 (72.7%)/3 (27.3%)	57 (85.1%)/10 (14.9%)	χ² = 4.83	0.185
Child Group, Children/Adults	17 (25.4%)/50 (74.6%)	14 (20.9%)/53 (79.1%)	3 (27.3%)/8 (72.7%)	17 (25.4%)/50 (74.6%)	χ² = 5.23	0.156

FIQ, full-scale IQ. Data are presented as mean ± SD or n (%).

## 4 Discussion

Across analyses, the present findings indicate that executive functioning contributes to social responsiveness in ASD through developmentally differentiated patterns, rather than through uniform EF–social associations across age groups. Specifically, when demographic and cognitive covariates were balanced, distinct executive components showed differential functional prominence in childhood versus adulthood, suggesting a reorganization in how executive demands are behaviorally expressed in social contexts. These results extend prior work by shifting the focus from whether EF is associated with social functioning to how different executive components assume relative functional roles across development. Interpreted at the level of behavioral expression, this pattern is consistent with developmental reorganization and compensatory processes, rather than global normalization of executive functioning.

Children with ASD demonstrated broad EF impairments, consistent with evidence linking executive abilities to social competence and adaptive functioning during early development (38). Early weaknesses in cognitive flexibility and working memory may constrain the acquisition of social reciprocity and communicative adaptability, thereby limiting opportunities for social learning (18, 39). Moreover, emotion regulation interacts with EF capacities in shaping social responsiveness, as difficulties in inhibitory control and cognitive shifting are associated with greater emotional dysregulation and social challenges (13, 40). In contrast, adults showed more differentiated EF profiles, with relative preservation in emotion regulation and self-monitoring. Importantly, these adult patterns were observed under covariate-balanced conditions, suggesting that the observed differentiation reflects a reorganization of behaviorally expressed executive demands rather than a global normalization of executive functioning. This pattern may reflect compensatory processes that enable some adults with ASD to maintain relatively effective regulation in everyday contexts (41). Continued prefrontal maturation may further support the recruitment of additional cognitive resources, potentially enabling adults with ASD to uphold aspects of social functioning despite residual inefficiencies (42). Rather than indicating a resolution of executive difficulties, these findings point to a developmental reorganization in which executive inefficiencies persist but become selectively expressed across domains at the behavioral level. At the level of everyday behavior, such selective efficiency may help adults manage social demands more effectively despite ongoing underlying inefficiencies, conceptually aligning with accounts of behavioral compensation or camouflaging, in which outwardly adaptive functioning may mask persistent neurocognitive difficulties (43). Finally, the lack of significant differences may also be influenced by the limited sensitivity of available measures to detect subtle deficits (44). Thus, EF difficulties in adulthood may reflect selectively expressed behavioral inefficiencies shaped by neural adaptation, strategic regulation, and measurement context, rather than the more globally expressed executive difficulties often observed in childhood.

The moderating analyses revealed that the relationship between EF and social responsiveness differed across developmental stages, with working memory showing a nominally stronger association in adults. This moderation effect is most consistent with a developmental shift in the relative functional weighting of executive components—such that working memory may assume greater prominence for social responsiveness in adulthood. This pattern suggests that as individuals mature, working memory becomes increasingly central to managing the complex cognitive and emotional demands of social interaction (45). For adults, maintaining conversational context, integrating subtle social cues, and regulating self-presentation likely depend on the continuous updating of social information. These findings are consistent with evidence that prefrontal systems supporting working memory and social cognition continue to mature and refine through adolescence and early adulthood, potentially supporting more strategic or compensatory regulation of social behavior in ASD (46, 47). Taken together, the moderation results highlight a shift in the functional weighting of executive components across development, rather than a uniform strengthening or weakening of executive influences on social behavior.

In children, the mediating effects of cognitive shifting and emotion regulation remained robust after accounting for demographic factors such as sex, age, and FIQ, suggesting that these EF contributions to social functioning are intrinsic to ASD neurocognitive mechanisms. Among the executive components examined in children, cognitive shifting showed a particularly strong mediating effect on social communication, consistent with evidence that cognitive flexibility facilitates reciprocal and contextually adaptive social behaviors in childhood ASD (39, 48). In adults, working memory exhibited a more pronounced mediating role, suggesting that as neurocognitive systems mature, different EF components contribute complementarily to social functioning (45).Taken together, these findings indicate that developmental differences in EF–social relationships are better conceptualized as a redistribution of functional roles across executive components, rather than as age-related changes in the overall strength of EF–social associations. Importantly, these patterns were observed after adjustment for demographic and cognitive factors, and all behavioral measures were expressed as standardized T-scores, indicating that the observed effects reflect relative within-group organization rather than absolute cross-age differences. Sample characteristics such as relatively high intellectual ability and gender imbalance may further shape how these EF–social mechanisms are behaviorally expressed, particularly through compensatory or adaptive strategies.

Latent profile analysis of EF and SRS T-scores identified four subgroups spanning a graded continuum of symptom severity, characterized by coordinated variation across executive functioning and social responsiveness. Prior research has documented substantial heterogeneity in executive functioning within ASD and other neurodevelopmental disorders, with developmental trajectories varying even among individuals sharing the same diagnosis (49, 50). A person-centered latent profile approach in children with ASD further demonstrated that subgroup membership can predict differential intervention responses (51). Consistent with these findings, our analysis revealed four EF–social responsiveness profiles reflecting systematic differences in overall severity, but also variation in the relative prominence of specific executive domains across profiles. Notably, cognitive shifting and social mannerisms showed the largest between-profile differentiation, whereas other executive domains exhibited more gradual increases across severity levels. These patterns suggest that, even within a broadly severity-ordered structure, distinct profiles may be characterized by differing configurations of executive and social features rather than symptom severity alone. Rather than prescribing specific intervention strategies, these profiles highlight the limitations of uniform assessment frameworks and underscore the importance of considering heterogeneity in the alignment between executive inefficiencies and social difficulties. From a clinical perspective, the identified profiles may be informative for risk stratification and for tailoring assessment focus, particularly in distinguishing individuals whose social challenges are closely coupled with executive inefficiencies from those whose profiles suggest alternative or additional contributing mechanisms. Such differentiation may support more individualized clinical formulation, without implying differential treatment efficacy based on cross-sectional behavioral data alone.

## 5 Limitations

This study has several limitations. First, the cross-sectional design restricts causal inferences regarding developmental trajectories, underscoring the need for longitudinal research to clarify developmental mechanisms over time. As a result, the observed developmental differences should be interpreted as reflecting differences in the organization and behavioral expression of executive–social relationships, rather than direct evidence of developmental change. Second, several sample-related factors should be considered. The adult ASD sample was relatively small and included small latent classes, particularly in the adult subgroup, which may have reduced statistical power for detecting moderation and mediation effects, and may have contributed to non-significant findings in some analyses. In addition, the sample exhibited a gender imbalance, which may limit the extent to which the observed patterns generalize across genders. Furthermore, the relatively high overall intellectual ability of the sample may constrain the generalizability of the findings to ASD populations with a broader range of cognitive functioning, particularly individuals with co-occurring intellectual disability or lower adaptive functioning. Third, although statistical strategies were used to control for normative differences, the use of different standardization norms for child and adult groups limits direct comparisons of absolute EF levels across developmental stages. Accordingly, the present findings are best interpreted in terms of relative within-group patterns and associations, rather than absolute cross-age differences in executive functioning. Importantly, information regarding prior clinical or educational intervention history was not available in the dataset. Intervention exposure may differ across age groups and individuals and may be more cumulative and heterogeneous in adulthood. Therefore, unmeasured intervention history could have influenced the observed executive–social profiles and cannot be ruled out as a contributor to the observed heterogeneity.

Future research should employ longitudinal designs with larger and more demographically balanced samples, as well as unified standardization procedures, to more precisely characterize the developmental mechanisms underlying cognitive–behavioral characteristics in ASD. In particular, studies with improved gender representation and a wider range of intellectual abilities will be important for determining the extent to which the present findings generalize across the autism spectrum.

## 6 Conclusions

This study shows that the association between executive functioning and social responsiveness in autism spectrum disorder is developmentally differentiated, with distinct executive components assuming different functional roles across childhood and adulthood. Cognitive flexibility was more central to social communication in childhood, whereas working memory showed greater relative prominence in adulthood. At the behavioral level, these findings suggest a developmental reorganization in the functional contribution of executive processes to social responsiveness, rather than uniform or globally impaired EF–social relationships across development. Latent profile analysis further highlighted substantial heterogeneity in EF–social coupling, underscoring individual variability and the limitations of uniform interpretive frameworks. Together, these results support a developmentally informed understanding of EF–social organization in ASD and highlight important practical implications for clinical support. Specifically, they suggest that the focus of intervention and clinical support may need to shift across development: during childhood, greater emphasis may be placed on supporting emotional reactivity and cognitive flexibility in social contexts, whereas in adulthood, clinical support may benefit from a stronger focus on metacognitive processes such as working memory. Rather than prescribing specific treatments, these findings offer a conceptual framework to inform developmentally tailored assessment and the alignment of intervention focus with stage-specific needs.

## Data Availability

The datasets presented in this study can be found in online repositories. The names of the repository/repositories and accession number(s) can be found below: http://fcon_1000.projects.nitrc.org/indi/abide/abide_II.html.
