# Supplementary material for: Age-related differences in the association between executive function and social responsiveness in autism spectrum disorder: a multi-method study
Source: Front Psychiatry. 2026 Mar 12;17:1729973. doi: 10.3389/fpsyt.2026.1729973 (PMC13017910; doi:10.3389/fpsyt.2026.1729973)
Supplement: Supplementary file 1 [file SupplementaryFile1.docx]

Supplementary Material

# Supplementary Data

## Methodological Supplement

### Descriptive statistics

Normality of all continuous variables was evaluated using the Shapiro–Wilk test within each age group (children and adults) and diagnostic group (ASD vs. Control). Because at least one subgroup violated the assumption of normality for most variables, all continuous data are reported as medians and interquartile ranges (IQR: Q1, Q3). Group differences in continuous variables were examined using Mann–Whitney U tests, and categorical variables were analyzed using chi-square tests. A two-tailed p value < 0.05 was considered statistically significant.

### Covariate Adjustment

We first conducted unadjusted between-group comparisons to examine raw differences in executive function (EF) and social responsiveness across age and diagnostic groups. Given theoretical associations of EF and social responsiveness with age, sex, and full IQ standard score (FIQ), subsequent analyses statistically controlled for these covariates. For the child sample, which had a larger sample size but imbalanced covariate distributions, entropy balancing was applied for preprocessing. Covariate balance was evaluated using standardized mean differences (SMD < 0.10). This approach reweighted control participants so that their weighted covariate distributions (mean and variance) matched those of the ASD group on age, sex, and FIQ. For the adult sample, due to a smaller size and potential instability of weighting estimates, covariate adjustment was performed using multiple regression analyses controlling for age, sex, and FIQ.

### Moderation and Moderated Mediation Analyses

To examine the moderating role of age group (children vs. adults) in the relationship between EF and social responsiveness, a two-stage analytical strategy was adopted. In stage 1, moderation analyses examined whether the association between EF and social responsiveness varied as a function of age group. A series of multiple regression models were constructed for each of the five EF subdomains and three social responsiveness subdomains, yielding a total of 15 models. Each model included the main effects of the EF subdomain, age group, and their two-way interaction (EF × Age Group), while controlling for the linear effects of age, sex, and FIQ. A statistically significant interaction would indicate that the relationship between EF and social responsiveness is moderated by age group. Bonferroni correction for multiple testing (15 comparisons) was applied, resulting in an adjusted significance threshold of α = 0.0033. Simple slope analyses were performed for each EF subdomain and each social responsiveness subdomain, separately for children and adults, to examine developmental differences in the EF–social responsiveness association. In stage 2, moderated mediation analyses were conducted to examine whether EF mediated the relationship between diagnostic group (ASD vs. Control) and SRS, and whether this mediation was moderated by age group. Age group was specified as a moderator of both the a-path (diagnostic group → EF) and b-path (EF → SRS). Sex and FIQ were included as covariates. Conditional indirect effects were estimated using bootstrap resampling (1,000 iterations), and mediation was considered significant when the 95% confidence interval did not include zero. No significant moderating or mediating effects were found, possibly reflecting the heterogeneity within the ASD population.

### Latent Profile Analysis (LPA)

To further explore this heterogeneity, a latent profile analysis (LPA) was conducted to identify data-driven subgroups of ASD based on EF and social responsiveness profiles. LPA was performed on standardized T-scores from five BRIEF subscales and three SRS subscales, ensuring that results reflected relative within-group patterns rather than absolute cross-age differences. Models specifying one to four latent classes were estimated sequentially. The upper limit of four classes was determined a priori based on sample size considerations. The present study included two age groups (children: n = 324; adults: n = 99). To ensure that each latent class contained a sufficient number of individuals within the smaller adult subsample—thereby maintaining parameter stability and interpretability—we decided not to fit models with more than four classes. Model comparison was primarily guided by information criteria, including the Akaike Information Criterion (AIC) and the Bayesian Information Criterion (BIC). The final number of classes was determined through a combination of statistical evidence and theoretical interpretability.

# Supplementary Figures and Tables

## Supplementary Figures

**Supplementary Figure 1. Covariate balance assessment before and after entropy balancing.**

##
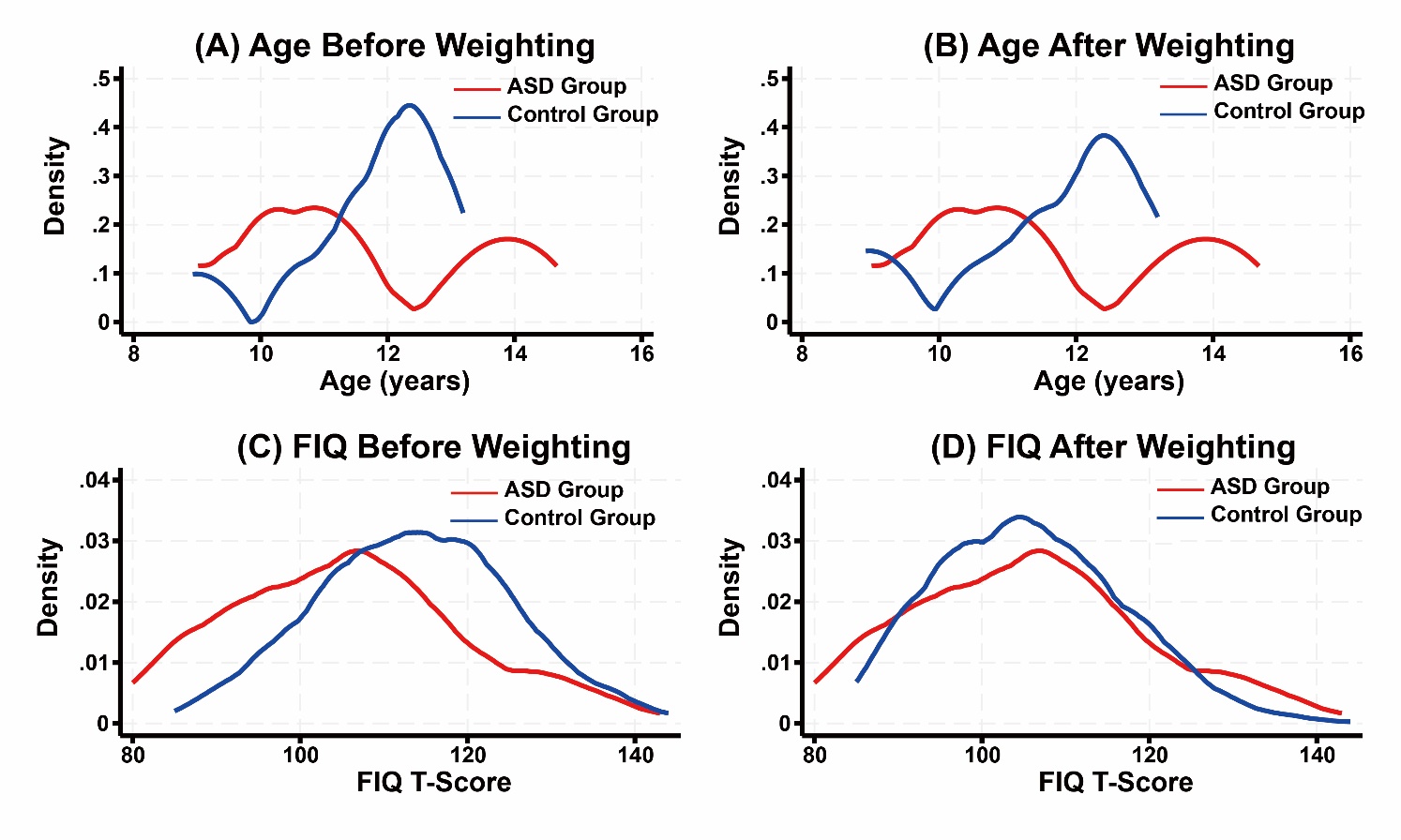


**Note:** Covariate balance assessment before and after entropy balancing. Density distributions illustrate the balance of age and FIQ between ASD and control groups. Red lines represent the ASD group (n = 134); blue lines represent the control group (n=190). (A) Age distribution before weighting shows baseline differences (standardized mean difference, SMD = -0.17). (B) Age distribution after entropy balancing demonstrates improved overlap (SMD = 0.00). (C) FIQ distribution before weighting indicates significant baseline differences (SMD = -0.63). (D) FIQ distribution after weighting shows successful alignment of distributions (SMD = 0.00). Entropy balancing effectively eliminated pre-existing covariate differences, with standardized mean differences improving from -0.17 to 0.00 for age and from -0.63 to 0.00 for FIQ, indicating successful covariate balance as further detailed in Supplementary Table 1.

## Supplementary Tables

**Supplementary Table 1. Comparison of baseline characteristics before and after application of entropy balancing weights in children sample.**

| **Variable** | **ASD Group (n = 134)** | **Control Group (n = 190)** | **P-value (Before)** | **Std. Diff (Before)** | **Std. Diff (After)** |
| --- | --- | --- | --- | --- | --- |
| Male, n (%) | 110 (82.09%) | 137 (72.11%) | 0.038 | 0.24 | 0.001 |
| Age, Mean (SD) | 11.50 (1.85) | 11.76 (1.19) | 0.124 | -0.17 | 0 |
| FIQ, Mean (SD) | 105.67 (14.02) | 113.58 (11.69) | <0.01 | -0.63 | 0 |

**Note:** Std. Diff = Standardized Difference, the primary metric for assessing between-group balance, where an absolute value less than 0.1 is generally considered negligible.

**Supplementary Table 2. Achieving covariate balance between ASD and control children’s groups through entropy balancing.**

|  |  | **ASD Group** | | | **Control Group** | | |
| --- | --- | --- | --- | --- | --- | --- | --- |
|  |  | **Mean** | **Variance** | **Skewness** | **Mean** | **Variance** | **Skewness** |
| Age | Before | 11.5 | 3.415 | 0.2908 | 11.76 | 1.415 | -1.193 |
|  | After | 11.5 | 3.415 | 0.2908 | 11.5 | 1.942 | -0.8023 |
| Sex | Before | 1.179 | 0.1481 | 1.674 | 1.279 | 0.2022 | 0.9858 |
|  | After | 1.179 | 0.1481 | 1.674 | 1.179 | 0.148 | 1.671 |
| FIQ | Before | 105.7 | 196.6 | 0.3297 | 113.6 | 136.8 | 0.0634 |
|  | After | 105.7 | 196.6 | 0.3297 | 105.7 | 121.9 | 0.3517 |

**Note:** This table presents the distributional characteristics (mean, variance, skewness) of covariates in the children ASD and control groups, before and after the application of entropy balancing weights. The data for the ASD group remain unchanged, as the weighting procedure was applied only to the control group to align its distribution with that of the ASD group. The entropy balancing algorithm successfully aligned the means of all covariates for the control group perfectly with those of the ASD group. Furthermore, the algorithm effectively adjusted the higher moments of the distributions (variance and skewness) in the control group, bringing them closer to the ASD distributions. Specifically, for the sex, its distribution was also perfectly matched. These results demonstrate that entropy balancing effectively eliminated not only the mean differences but also significantly reduced the differences in distributional shapes, thereby substantially mitigating potential confounding bias.

**Supplementary Table 3. Distribution of entropy balancing weights and effective sample size.**

| **Weights Statistics** | **Control Group** | **ASD Group** |
| --- | --- | --- |
| Mean | 0.71 | 1 |
| Standard Deviation | 0.55 | 0 |
| Minimum | 0.10 | 1 |
| Maximum | 3.42 | 1 |
| Effective Sample Size (ESS) | 117 | 134 |

**Note:** The entropy balancing algorithm assigned a fixed weight of 1 to the ASD group and generated the above weights for the control group to balance the distributions of age, sex, and FIQ. The ESS was calculated as $\frac{\left( \sum weight \right)^{2}}{\sum\left( {weight}^{2} \right)}$. The distribution of weights (SD = 0.55, range = 0.10 - 3.42 for the control group) indicates that a subset of control units was assigned higher weights to achieve precise covariate balance. The ESS of 117 for the weighted control group indicates a reduction in statistical precision compared to the original sample, which is a common trade-off in weighting methodologies to eliminate confounding bias.

**Supplementary Table 4. Multiple linear regression analyses of EF and social responsiveness in adult sample: standardized coefficients.**

| **Variable** | **Group (β)** | **Sex (β)** | **Age (β)** | **FIQ (β)** | **Adjust R²** |
| --- | --- | --- | --- | --- | --- |
| SRS-Total | 0.710 ^* * *^ | 0.09 | 0.18 | 0.00 | 0.70 |
| SRS-Communication | 0.669 ^* * *^ | 0.11 | 0.21 | 0.03 | 0.66 |
| SRS-Mannerisms | 0.782 ^* * *^ | -0.128 ^*^ | 0.09 | 0.02 | 0.67 |
| Inhibit | 0.837 ^* * *^ | 0.17 | 0.23 | 0.03 | 0.34 |
| Shift | 0.655 ^* * *^ | 0.02 | 0.10 | 0.01 | 0.52 |
| Emotional Control | 0.451 ^*^ | 0.00 | 0.13 | 0.08 | 0.29 |
| Working Memory | 0.458 ^* *^ | -0.169 ^*^ | 0.29 | 0.09 | 0.47 |
| Monitor | 0.339 ^*^ | 0.09 | .385 ^*^ | 0.04 | 0.44 |

**Note:** Table presents standardized regression coefficients (β). *p < 0.05, **p < 0.01, ***p< 0.001. The reference group for the analysis is the control group for group comparison and female for sex comparison. Adjusted R² represents the proportion of variance in the dependent variable explained by the full model, adjusted for the number of predictors to account for model complexity.

**Supplementary Table 5.** **Partial η² effect sizes for EF and social responsiveness in the adult sample.**

| **Variable** | **Group** **Partial η²** | **Sex Partial η²** | **Age Partial η²** | **FIQ Partial η²** | **Adjusted R²** |
| --- | --- | --- | --- | --- | --- |
| SRS-Total | 0.26 | 0.02 | 0.02 | 0.00 | 0.70 |
| SRS-Communication | 0.22 | 0.03 | 0.03 | 0.00 | 0.66 |
| SRS-Mannerisms | 0.28 | 0.04 | 0.01 | 0.00 | 0.67 |
| Inhibit | 0.19 | 0.03 | 0.02 | 0.00 | 0.34 |
| Shift | 0.16 | 0.00 | 0.00 | 0.00 | 0.52 |
| Emotional Control | 0.06 | 0.00 | 0.01 | 0.01 | 0.29 |
| Working Memory | 0.08 | 0.04 | 0.04 | 0.02 | 0.47 |
| Monitor | 0.04 | 0.01 | 0.06 | 0.00 | 0.44 |

**Note:** Partial η² represents the unique effect size of each predictor after controlling for other variables. Adjusted R² indicates the proportion of variance explained by the full model, adjusted for the number of predictors.

**Supplementary Table 6. Multicollinearity diagnostics for adult group multiple regression analyses (Variance inflation factors, VIF).**

| **Predictor** | **VIF** | **Tolerance (1/VIF)** | **Interpretation** |
| --- | --- | --- | --- |
| Group | 4.81 | 0.21 | Acceptable |
| Age | 4.41 | 0.23 | Acceptable |
| Sex | 1.2 | 0.83 | Good |
| FIQ | 1.03 | 0.97 | Excellent |

**Note:** Conventional thresholds: VIF < 5 indicate acceptable; VIF < 2 indicate ideal. Tolerance values > 0.20 indicate minimal multicollinearity concerns. All VIF values were below 5, indicating that multicollinearity does not substantially affect the regression results. VIF values were consistent across all models due to identical independent variable sets.

**Supplementary Table 7. VIF for moderated regression analyses.**

| **EF** | **Interaction VIF** | **Group VIF** | **Predictor VIF** | **Mean VIF** | **Interpretation** |
| --- | --- | --- | --- | --- | --- |
| Inhibit | 1.54 | 1.39 | 1.32 | 1.42 | Excellent |
| Shift | 1.87 | 1.62 | 1.47 | 1.65 | Excellent |
| Emotional Control | 1.59 | 1.37 | 1.36 | 1.44 | Excellent |
| Working Memory | 1.71 | 1.57 | 1.46 | 1.58 | Excellent |
| Monitor | 1.71 | 1.53 | 1.44 | 1.56 | Excellent |

**Note:** Conventional thresholds: VIF < 5 indicate acceptable; VIF < 2 indicate ideal. VIF values were identical across different SRS subscales within each EF domain. All VIF values were below 5, indicating no multicollinearity concerns.

**Supplementary Table 8.** **Model fit indices for LPA models (1-4 Classes).**

| **Model** | **AIC** | **BIC** |
| --- | --- | --- |
| 1-Class | 4201.333 | 4252.772 |
| 2-Class | 3736.853 | 3817.226 |
| 3-Class | 3587.947 | 3697.254 |
| 4-Class | 3472.408 | 3610.651 |

**Note:** Model selection was guided by information criteria, with the four-class solution yielding the lowest AIC (3472.41) and BIC (3610.65) values, reflecting the optimal balance between model fit and parsimony.

**Supplementary Table 9.** **Classification quality metrics.**

| **Class** | **n (%)** | **Average Posterior Probability** | **Posterior Probability Range** |
| --- | --- | --- | --- |
| 1 | 39 (21.20%) | 0.95 | 0.73 - 1.00 |
| 2 | 67 (36.41%) | 0.95 | 0.53 - 1.00 |
| 3 | 67 (36.41%) | 0.96 | 0.60 - 1.00 |
| 4 | 11 (5.98%) | 0.97 | 0.86 - 1.00 |

**Note:** Classification quality indicates excellent assignment certainty (> 0.80 considered good, > 0.90 considered excellent).

**Supplementary Table 10.** **Effect sizes for pairwise class comparisons.**

| **Comparison** | **Cohen's d** | **Effect Size Interpretation** |
| --- | --- | --- |
| Class 2 vs Class 1 | 2.39 | Very Large |
| Class 3 vs Class 1 | 5.04 | Very Large |
| Class 4 vs Class 1 | 6.02 | Very Large |
| Class 3 vs Class 2 | 2.9 | Very Large |
| Class 4 vs Class 2 | 5.2 | Very Large |
| Class 4 vs Class 3 | 2.84 | Very Large |

**Note:** Effect size magnitudes were classified as small (d = 0.2), medium (d = 0.5), and large (d = 0.8) per Cohen's standards.
